# CD28^−^ and CD28^low^CD8^+^ Regulatory T Cells: Of Mice and Men

**DOI:** 10.3389/fimmu.2017.00031

**Published:** 2017-01-23

**Authors:** Yirajen Vuddamalay, Joost P. M. van Meerwijk

**Affiliations:** ^1^School of Health Sciences, University of Technology, Port Louis, Mauritius; ^2^Institut National de la Santé et de la Recherche Médicale (INSERM), U1043, Toulouse, France; ^3^Centre National de la Recherche Scientifique (CNRS), U5282, Toulouse, France; ^4^Université de Toulouse, Université Paul Sabatier, Centre de Physiopathologie de Toulouse Purpan (CPTP), Toulouse, France

**Keywords:** tolerance, regulatory T cells, CD8^+^ T-lymphocytes, human, mouse, thymus, immunoregulation

## Abstract

Since the rebirth of regulatory (formerly known as suppressor) T cells in the early 1990s, research in the field of immune-regulation by various T cell populations has quickly gained momentum. While T cells expressing the transcription factor Foxp3 are currently in the spotlight, several other T cell populations endowed with potent immunomodulatory capacities have been identified in both the CD8^+^ and CD4^+^ compartment. The fundamental difference between CD4^+^ and CD8^+^ T cells in terms of antigen recognition suggests non-redundant, and perhaps complementary, functions of regulatory CD4^+^ and CD8^+^ T cells in immunoregulation. This emphasizes the importance and necessity of continuous research on both subpopulations of regulatory T cells (Tregs) so as to decipher their complex physiological relevance and possible synergy. Two distinct CD8-expressing Treg populations can be distinguished based on expression of the co-stimulatory receptor CD28. Here, we review the literature on these (at least in part) thymus-derived CD28^low^ and peripherally induced CD28^−^CD8^+^ Tregs.

## Introduction

The prerequisite to the prevention of immunopathologies such as autoimmunity and chronic inflammation is the maintenance of an immune homeostasis that relies mainly on intricate mechanisms of tolerance to self and innocuous non-self antigens. Through their multifaceted actions, regulatory T cells (Tregs) play an unparalleled role in modulating both innate and adaptive responses. As such, Tregs prevent autoimmune disorders, control immune reactions at environmental surfaces, modulate anti-infectious responses, and contribute to fetomaternal tolerance [reviewed in Ref. (1–3)].

Historically speaking, the first suppressor population to be described were T cells expressing the CD8 co-receptor. Indeed, the T cell population identified by Cantor et al., which act in an antigen-specific manner to suppress immune reactions, expressed the surface marker Lyt2, now known as CD8α (4, 5). Since then, the field has had its fair share of whirls on the wheel of scientific (mis)fortune. The concatenation of events from the downfall of suppressor T cells to its rebirth (or rebranding) as Tregs have extensively been reviewed elsewhere (6–8) and will not be discussed here.

The advent of molecular immunology in the postsuppressor era unequivocally established the T cell population expressing the forkhead/winged helix transcription factor Foxp3 as a key player in the fine regulation of immune responses [reviewed in Ref. (9)]. Indeed, in mice, invalidating mutations in the *Foxp3* gene or specific ablation of Foxp3^+^ T cells lead to the development of a fatal lymphoproliferative disorder (10–13) and humans with mutations in the FOXP3 gene suffer from the lethal immune-dysregulation polyendocrinopathy enteropathy X linked syndrome (14, 15). In parallel, several other regulatory CD4^+^ and CD8^+^ subsets have been identified and characterized in both mice and humans (16–20). Distinct Treg (sub)populations differ in their origin, development, and mechanisms of action which *in fine* define their physiological role. As such, determining the specific function of a given Treg population mandates extensive research to identify the different molecular and cellular factors that govern its existence. We and others have contributed to unveil some key features of the CD8-expressing Treg population that is characterized by the expression of low levels of the co-stimulatory molecule CD28; CD8^+^CD28^low^ Treg.

## CD8^+^CD28^low^ Treg in Mice

The immunosuppressive capacity of CD8^+^CD28^low^ was first described in a murine model of multiple sclerosis. Najafian et al. showed that CD8 knockout (CD8 KO) mice were more susceptible to the induction of experimental autoimmune encephalomyelitis (EAE) than wild-type (WT) mice suggesting a protective effect of CD8^+^ cells. Adoptive transfer of CD8^+^CD28^low^ T cells from WT animals into CD8 KO recipients significantly reduced the severity of the disease. No such decrease was observed with the adoptive transfer of CD8^+^CD28^high^ T cells. Furthermore, CD8^+^CD28^low^ T cells but not their CD28^high^ counterpart could suppress *in vitro* the production of interferon-γ by CD4^+^ T cells specific for the myelin oligodendrocyte glycoprotein used to induce EAE. The suppressive function of the CD8^+^CD28^low^ Treg required an interaction with antigen-presenting cells (APC), which led to the downregulation of CD80, CD86, and CD40 expression on the APC (21). In a similar model, Yang et al. have shown that pretreatment of mice with a group of 15-amino acid-long trichosanthin-derived peptides reduced the clinical score of EAE as compared to untreated animals. Attenuation of the disease was attributed to the expansion and activation of IL10-producing-CD8^+^CD28^low^ Treg (22).

Previous work by our team has shown that CD8^+^CD28^low^ Treg can prevent intestinal inflammation in a well-established experimental colitis model where pathology is induced by the adoptive transfer of naïve CD4^+^CD45RB^high^ T cells into lymphopenic animals [recombinase activating gene 2 (RAG2) deficient or severe combined immunodeficiency mice (23, 24)]. Cotransfer of freshly isolated splenic CD8^+^CD28^low^ T cells from WT animals with the colitogenic cells prevented onset of colitis. Similar results were obtained with CD8^+^CD28^low^ T cells isolated from the lamina propria of the intestine (25). These CD8αβ^+^CD28^low^ Treg expressed a large repertoire of the TCRαβ heterodimer (26). Protection from colitis was dependent on IL-10 production by the Treg and on the responsiveness of the colitogenic T-cells to transforming growth factor β (TGF-β), underlining the non-redundant functions of these two immunomodulatory cytokines in the control of intestinal inflammation by CD8^+^CD28^low^ Treg (25). Importantly, in contrast to CD4^+^CD25^high^ Treg, CD8^+^CD28^low^ Treg from unmanipulated mice do not express the transcription factor Foxp3. More recently, in mice immunized with ovalbumin and subsequently intranasally challenged with ovalbumin encased in oligomannose-coated liposomes, an expansion of CD8^+^CD28^low^ (and CD4^+^Foxp3^+^) Treg was observed. Upon adoptive transfer, the CD8^+^CD28^low^ Treg reduced the severity of allergic diarrhea (27).

## Autoimmune Regulator (AIRE) and the Development of CD8^+^CD28^low^ Treg

The transcription factor AIRE is primarily expressed by medullary epithelial cells of the thymus (mTEC) where it controls cellular maturation and the ectopic expression of thousands of tissue-specific antigens (28, 29). Presentation of these peripheral antigens by mTEC leads to the negative selection of auto-specific conventional T cells (30–32). Furthermore, AIRE modulates the production of chemokines by mTEC, involved in the migration of thymocytes and dendritic cells from the cortex to the medulla in the thymus (33, 34). As such, AIRE is a key regulator of central tolerance. Indeed, loss-of-function mutations in the AIRE gene lead to the autoimmune polyendocrinopathy candidiasis ectodermal dystrophy (APECED) syndrome also known as APS for autoimmune polyglandular syndrome (35, 36). While chronic mucocutaneous candidiasis, hypoparathyroidism, and hypoadrenalism are considered to be the classic triad hallmarks of this autoimmune syndrome (37), about 25% of APECED patients are also affected by gastrointestinal diseases ranging from chronic diarrhea and obstipation (38). In children suffering from APECED, these intestinal ailments can lead to malabsorption, various deficiencies, growth impairment, and even death (39, 40). Importantly, some do even consider gastrointestinal symptoms to be the first manifestation of APECED (38). Mice deficient for AIRE also exhibit (though to a lesser extent) autoimmune symptoms such as presence of autoantibodies and cellular infiltration in various organs (41). Since CD8^+^CD28^low^ Treg can efficiently prevent intestinal inflammation, a prominent symptom in APECED, the potential role of AIRE in the development of this Treg population was evaluated.

Our comparative study of CD8^+^CD28^low^ Treg from WT and AIRE-deficient (AIRE KO) mice revealed that while both Treg populations were present in similar proportions and exhibited comparable immunosuppressive activity *in vitro*, Treg from AIRE KO animals failed to prevent intestinal inflammation in the colitis model (26). Gene expression patterns, cell-surface marker expression, IL-10 production, and *in vitro* suppressive capacity of WT and AIRE KO CD8^+^CD28^low^ Treg were indistinguishable. However, a small difference was found between the T-cell receptor (TCR) repertoires expressed by WT vs. KO Treg. Based on these observations, we concluded that AIRE is involved in shaping the TCR-repertoire of CD8^+^CD28^low^ Treg. To our knowledge, this was the first definite demonstration that a deficiency in AIRE leads to the functional defect of a Treg population. This pioneer study is in line with more recent studies that have provided molecular evidence, through TCR repertoire analysis, that AIRE is essential for the thymic development of CD4^+^Foxp3^+^ Treg with unique individual TCRs (42–44). Taken together, these studies have established that AIRE not only drives the negative selection of conventional T cells but is also involved in the differentiation of CD8^+^ and CD4^+^ Treg populations.

## Origin of CD8^+^CD28^low^ Treg

**Figure 1:**
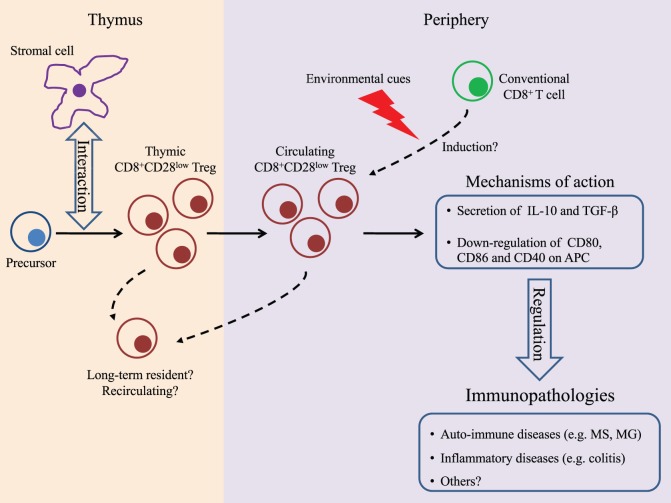
**Summary of findings on CD8^+^CD28^low^ regulatory T cells (Tregs)**. In the thymus, T cell precursors will interact with stromal cells presenting antigens that are expressed under control of the transcription factor autoimmune regulator and differentiate into tTreg. In the periphery, these cells will enforce their regulatory function by secreting immunomodulatory cytokines and/or by inhibiting antigen-presenting cells. It cannot be excluded that CD8^+^CD28^low^ Treg can also differentiate, under specific tolerogenic conditions, in the periphery. So far, the immunosuppressive capacity of CD8^+^CD28^low^ Tregs has been documented in experimental models of multiple sclerosis, myasthenia gravis, and colitis. Solid lines indicate established features, and dashed lines indicate potential characteristics.

Based on our current understanding of the development of CD4^+^Foxp3^+^ Treg, it is commonly accepted that Treg in general can have two distinct origins: intrathymic development of “tTreg” from hematopoietic precursors and extrathymic (or peripheral) differentiation of “pTreg” from conventional T cells given appropriate environmental cues [reviewed in Ref. (45, 46)]. Since data from the literature have attributed distinct singular functions to tTreg and pTreg (47–49), the identification of the origin of CD8^+^CD28^low^ Treg was an important milestone in the quest to better characterize this population. Our observation that AIRE, which is primarily expressed in the thymus, is involved in the development of the CD8^+^CD28^low^ Treg repertoire suggested a thymic origin for CD8^+^CD28^low^ Treg. However, expression of AIRE has also been reported in both hematopoietic and stromal lineages outside of the thymus (50–52). Importantly, these extrathymic AIRE-expressing cells have tolerogenic properties (53) and thus in theory may induce differentiation of conventional T cells into Tregs. We recently demonstrated that mature CD4^−^CD8^+^TCR^high^ thymocytes expressing low levels of CD28, isolated from WT mice, can efficiently suppress the *in vitro* proliferation of CD4^+^ T cells (54). However, since T cells including Tregs can recirculate from the periphery back to the thymus (55, 56), their presence in this primary lymphoid organ was not sufficient to confirm their origin. Definite proof of the thymic origin of CD8^+^CD28^low^ Treg came from the analysis of transgenic mice expressing the green fluorescent protein (GFP) under the control of the RAG2 promoter [RAG–GFP mice, Ref. (57)]. In the thymus, thymocytes express RAG2 at the early stages of their development and then terminate its expression after positive selection (58). As such, in RAG–GFP animals, the GFP protein whose expression parallels that of RAG2 and has a half-life of 56 h serves as a molecular marker for lymphocyte aging in the thymus allowing for the discrimination between “freshly” developed mature T cells that express GFP and recirculating T cells that do not (59). Analysis of RAG–GFP mice revealed that while approximately 20% of mature thymic CD8^+^CD28^low^ T cells are deprived of GFP expression (i.e., recirculating or long-term thymus resident cells), the major proportion of this T cell population are newly developed cells. Importantly, the GFP^+^ compartment of the mature thymic CD8^+^CD28^low^ T cells demonstrated immunosuppressive activity *in vitro* hence firmly establishing the thymic origin of CD8^+^CD28^low^ Treg in mice (54). However, the interesting possibility that the pool of circulating CD8^+^CD28^low^ Treg may be composed of both tTreg and pTreg must also be considered. Indeed, in an experimental model of myasthenia gravis (MG), exposure to specific antigens (the dual-altered peptide) led to the emergence of CD8^+^CD28^low^ Treg (60). While it can be argued that the emergence of Treg could be due to the expansion of preexisting tTreg, the alternate hypothesis of an induction of *bona fide* pTreg cannot be excluded (Figure 1).

## CD8^+^CD28^low^ Treg in Humans

A population of CD8^+^CD28^low^ T cell exhibiting similar immunosuppressive characteristics as its murine homolog has recently been identified in humans. Analysis of peripheral blood mononuclear cells (PBMCs) has revealed a substantial percentage (between 10 and 13%) of CD28^low^-expressing cells among naive CD8^+^ T cells. Importantly, following *in vitro* activation, these cells produce the same cytokines (i.e., IL-10 and TGF-β), which confer CD8^+^CD28^low^ Treg their immunomodulatory ability in experimental mouse models. Similar results were obtained when human thymii isolated from children aged from 0 to 10 years were analyzed (54). Taken together, these results from human studies strongly suggest that, similar to mouse, CD8^+^CD28^low^ T cell endowed with immunosuppressive capacity are present in human PBMCs and that they develop in the human thymus.

## CD8^+^CD28^−^ Treg in Humans and Mice

Based on CD28 expression, another Treg population has previously been described in humans. Cyclic stimulations of PBMCs with allogenic APC induced CD8^+^ T cells deprived of CD28 expression, which inhibited cellular proliferation in these *in vitro* cultures (61). Since then, several groups have tried to develop, with more or less success, their own strategies to induce CD8^+^CD28^−^ Treg *in vitro* by stimulating PBMCs with cocktails of cytokines in the presence or absence of antigens (62, 63), with phorbol12-myristate 13-acetate/ionomycin or phytohemagglutinin (64) or with a recombinant immunoglobulin-like transcript 3 (ILT3)-Fc fusion protein (65, 66). CD8^+^CD28^−^ Tregs express GITR, CD25, CD103, CD62L, and 4-IBB and are MHC class I restricted (67). They exert their immunomodulatory activity by inducing the expression of ILT3 and ILT4 on dendritic cells, thus rendering them tolerogenic (66). Intriguingly, human mesenchymal stromal cells have recently been shown to enhance the immunomodulatory function of CD8^+^CD28^−^ Treg by reducing their rate of apoptosis (68).

CD8^+^CD28^−^ T cells with a regulatory phenotype have been observed in patients having undergone successful organ transplantation (69–71), alloanergized HLA-mismatched bone marrow graft (72), and allogenic platelet transfusion (73) or suffering from autoimmune diseases (74–76), pregnancy complications (77), and cancers (78–80). Importantly, CD8^+^CD28^−^ T cells isolated from healthy donors are not immunosuppressive (69). Hence, it would seem that CD8^+^CD28^−^ Treg are induced in the periphery following disturbances of the immune homeostasis.

A mouse homolog of human CD8^+^CD28^−^ pTreg may also exist. Ben-David et al. showed that in an experimental model of MG where pathology is triggered by immunization with a myasthenogenic peptide, injection of a dual-altered peptide induces the emergence of CD8^+^CD28^−^ Treg that efficiently suppress the autoimmune response. Flow cytometry analysis of these cells suggested that these Tregs may express low levels of Foxp3 (60).

## CD28^−^ vs. CD28^low^CD8^+^ Treg in Humans and Mice

Najafian et al. initially showed that total CD8^+^ T cells isolated from CD28-deficient mice (i.e., CD8^+^CD28^−^ cells) exhibited immunosuppressive activity *in vitro* and decreased the severity of EAE in adoptive transfer experiments. However, the CD8^+^ T cells isolated from WT mice that inhibited severity of EAE clearly expressed low levels of CD28 (21). In our initial report on the prevention of experimental colitis in the mouse, the CD8^+^ Treg, which we inaccurately termed CD28^−^, also clearly expressed low but detectable levels of CD28. In unmanipulated specific pathogen-free WT mice, we only observed subsets of CD8^+^ T cells expressing low or high levels of CD28 but none that are deprived of expression of this co-stimulatory molecule (25, 26, 54). In humans, their low but readily detectable level of expression of CD28, their presence in the thymus, and their naive phenotype clearly distinguish CD8^+^CD28^low^ Treg from CD8^+^CD28^−^ Treg that do not express CD28 at levels exceeding background, are not found in the thymus, and have an activated phenotype (54). We therefore conclude that the co-stimulatory molecule CD28 allows for the identification of two distinct CD8^+^ subsets: CD28^low^ tTreg and CD28^−^ p Treg.

## Concluding Remarks

While the various studies discussed here have helped to decipher key features of CD8^+^CD28^low^ T cells and in parallel establish them as a potent Treg population in both mice and humans, several burning questions concerning these Treg remain unanswered, the most important one being perhaps their biological function(s) under homeostatic and pathologic conditions. We believe that the identification of other, more discriminative, markers of CD8^+^CD28^low^ Treg will greatly help in achieving this goal. Currently, this Treg population can only be characterized by their low levels of expression of CD28 allowing for only a minimal estimation of their proportions by flow cytometry analysis (25, 26, 54). Furthermore, the absence of a better marker is hindering a panoply of key experiments such as specific localization in tissues and lymphoid organs, antibody-specific depletion, germline, and/or conditional knockout strategies.

Up till now, research on CD8^+^CD28^low^ Treg had been confined to murine studies (21, 25, 26). Even though the potent immunoregulatory capacity of CD8^+^CD28^low^ has been documented in these experimental models of inflammation, its relevance in human diseases remains unknown. In parallel, defects in various CD4^+^ and CD8^+^ Treg populations have been reported in human autoimmune diseases and immune-mediated inflammatory pathologies (81–87). The identification of CD8^+^CD28^low^ Treg in humans is hence paving the way to further studies so as to gain insight into the physiological function of this Treg population and its potential involvement in human pathologies.

## Author Contributions

YV and JPMvM designed the outline and wrote the manuscript.

## Conflict of Interest Statement

The authors declare that the research was conducted in the absence of any commercial or financial relationships that could be construed as a potential conflict of interest.

## Abbreviations

AIRE, autoimmune regulator; APC, antigen-presenting cells; APECED, autoimmune polyendocrinopathy candidiasis ectodermal dystrophy; APS, autoimmune polyglandular syndrome; EAE, experimental autoimmune encephalomyelitis; Foxp3, forkhead/winged helix transcription factor; GFP, green fluorescent protein; IFN-γ, interferon-γ; IPEX, immune dysregulation polyendocrinopathy enteropathy X linked; ILT, immunoglobulin-like transcript; RAG, recombinase activating gene; SCID, severe combined immunodeficiency; TGF-β, transforming growth factor β.

## References

[1] Peterson RA. Regulatory T-cells: diverse phenotypes integral to immune homeostasis and suppression. Toxicol Pathol (2012) 40(2):186–204. 10.1177/0192623311430693 22222887

[2] Ruocco MG Chaouat G Florez L Bensussan A Klatzmann D. Regulatory T-cells in pregnancy: historical perspective, state of the art, and burning questions. Front Immunol (2014) 5:389. 10.3389/fimmu.2014.00389 25191324 PMC4139600

[3] Shevach EM. Biological functions of regulatory T cells. Adv Immunol (2011) 112:137–76. 10.1016/B978-0-12-387827-4.00004-8 22118408

[4] Cantor H Shen FW Boyse EA. Separation of helper T cells from suppressor T cells expressing different Ly components. II. Activation by antigen: after immunization, antigen-specific suppressor and helper activities are mediated by distinct T-cell subclasses. J Exp Med (1976) 143(6):1391. 10.1084/jem.143.6.1391 1083888 PMC2190222

[5] Jandinski J Cantor H Tadakuma T Peavy DL Pierce CW. Separation of helper T cells from suppressor T cells expressing different Ly components. I. Polyclonal activation: suppressor and helper activities are inherent properties of distinct T-cell subclasses. J Exp Med (1976) 143(6):1382–90. 10.1084/jem.143.6.1382 1083887 PMC2190204

[6] Germain RN. Special regulatory T-cell review: a rose by any other name: from suppressor T cells to Tregs, approbation to unbridled enthusiasm. Immunology (2008) 123(1):20–7. 10.1111/j.1365-2567.2007.02779.x 18154615 PMC2433291

[7] Kapp JA. Special regulatory T-cell review: suppressors regulated but unsuppressed. Immunology (2008) 123(1):28–32. 10.1111/j.1365-2567.2007.02773.x 18154616 PMC2433289

[8] Waldmann H. Special regulatory T cell review: the suppression problem! Immunology (2008) 123(1):11–2. 10.1111/j.1365-2567.2007.02776.x 18154612 PMC2433285

[9] Ramsdell F Ziegler SF. FOXP3 and scurfy: how it all began. Nat Rev Immunol (2014) 14(5):343–9. 10.1038/nri3650 24722479

[10] Fontenot JD Gavin MA Rudensky AY. Foxp3 programs the development and function of CD4+CD25+ regulatory T cells. Nat Immunol (2003) 4(4):330–6. 10.1038/ni904 12612578

[11] Hori S Nomura T Sakaguchi S. Control of regulatory T cell development by the transcription factor Foxp3. Science (2003) 299(5609):1057–61. 10.1126/science.1079490 12522256

[12] Khattri R Cox T Yasayko SA Ramsdell F. An essential role for Scurfin in CD4+CD25+ T regulatory cells. Nat Immunol (2003) 4(4):337–42. 10.1038/ni909 12612581

[13] Kim JM Rasmussen JP Rudensky AY. Regulatory T cells prevent catastrophic autoimmunity throughout the lifespan of mice. Nat Immunol (2007) 8(2):191–7. 10.1038/ni1428 17136045

[14] Bennett CL Christie J Ramsdell F Brunkow ME Ferguson PJ Whitesell L The immune dysregulation, polyendocrinopathy, enteropathy, X-linked syndrome (IPEX) is caused by mutations of FOXP3. Nat Genet (2001) 27(1):20–1. 10.1038/83713 11137993

[15] Powell BR Buist NR Stenzel P. An X-linked syndrome of diarrhea, polyendocrinopathy, and fatal infection in infancy. J Pediatr (1982) 100(5):731–7. 10.1016/S0022-3476(82)80573-8 7040622

[16] Geginat J Paroni M Facciotti F Gruarin P Kastirr I Caprioli F The CD4-centered universe of human T cell subsets. Semin Immunol (2013) 25(4):252–62. 10.1016/j.smim.2013.10.012 24183700

[17] Pankratz S Ruck T Meuth SG Wiendl H. CD4(+)HLA-G(+) regulatory T cells: molecular signature and pathophysiological relevance. Hum Immunol (2016) 77(9):727–33. 10.1016/j.humimm.2016.01.016 26826445

[18] Pomie C Menager-Marcq I van Meerwijk JP. Murine CD8+ regulatory T lymphocytes: the new era. Hum Immunol (2008) 69(11):708–14. 10.1016/j.humimm.2008.08.288 18817827

[19] Tsai S Clemente-Casares X Santamaria P CD8(+) Tregs in autoimmunity: learning “self”-control from experience. Cell Mol Life Sci (2011) 68(23):3781–95. 10.1007/s00018-011-0738-y 21671120 PMC11114820

[20] Zeng H Zhang R Jin B Chen L Type 1 regulatory T cells: a new mechanism of peripheral immune tolerance. Cell Mol Immunol (2015) 12(5):566–71. 10.1038/cmi.2015.44 26051475 PMC4579656

[21] Najafian N Chitnis T Salama AD Zhu B Benou C Yuan X Regulatory functions of CD8+CD28- T cells in an autoimmune disease model. J Clin Invest (2003) 112(7):1037–48. 10.1172/JCI17935 14523041 PMC198520

[22] Yang N Li Z Jiao Z Gu P Zhou Y Lu L A trichosanthin-derived peptide suppresses type 1 immune responses by TLR2-dependent activation of CD8(+)CD28(-) Tregs. Clin Immunol (2014) 153(2):277–87. 10.1016/j.clim.2014.05.005 24858261

[23] Morrissey PJ Charrier K Braddy S Liggitt D Watson JD. CD4+ T cells that express high levels of CD45RB induce wasting disease when transferred into congenic severe combined immunodeficient mice. Disease development is prevented by cotransfer of purified CD4+ T cells. J Exp Med (1993) 178(1):237–44. 10.1084/jem.178.1.237 8100269 PMC2191069

[24] Powrie F Leach MW Mauze S Caddle LB Coffman RL. Phenotypically distinct subsets of CD4+ T cells induce or protect from chronic intestinal inflammation in C. B-17 scid mice. Int Immunol (1993) 5(11):1461–71. 10.1093/intimm/5.11.1461 7903159

[25] Menager-Marcq I Pomie C Romagnoli P van Meerwijk JP. CD8+CD28- regulatory T lymphocytes prevent experimental inflammatory bowel disease in mice. Gastroenterology (2006) 131(6):1775–85. 10.1053/j.gastro.2006.09.008 17087950 PMC1950262

[26] Pomie C Vicente R Vuddamalay Y Lundgren BA van der Hoek M Enault G Autoimmune regulator (AIRE)-deficient CD8+CD28low regulatory T lymphocytes fail to control experimental colitis. Proc Natl Acad Sci U S A (2011) 108(30):12437–42. 10.1073/pnas.1107136108 21746930 PMC3145727

[27] Kawakita A Shirasaki H Yasutomi M Tokuriki S Mayumi M Naiki H Immunotherapy with oligomannose-coated liposomes ameliorates allergic symptoms in a murine food allergy model. Allergy (2012) 67(3):371–9. 10.1111/j.1398-9995.2011.02777.x 22423374

[28] Derbinski J Gabler J Brors B Tierling S Jonnakuty S Hergenhahn M Promiscuous gene expression in thymic epithelial cells is regulated at multiple levels. J Exp Med (2005) 202(1):33–45. 10.1084/jem.20050471 15983066 PMC2212909

[29] Johnnidis JB Venanzi ES Taxman DJ Ting JP Benoist CO Mathis DJ. Chromosomal clustering of genes controlled by the aire transcription factor. Proc Natl Acad Sci U S A (2005) 102(20):7233–8. 10.1073/pnas.0502670102 15883360 PMC1129145

[30] Anderson MS Venanzi ES Chen Z Berzins SP Benoist C Mathis D. The cellular mechanism of Aire control of T cell tolerance. Immunity (2005) 23(2):227–39. 10.1016/j.immuni.2005.07.005 16111640

[31] DeVoss J Hou Y Johannes K Lu W Liou GI Rinn J Spontaneous autoimmunity prevented by thymic expression of a single self-antigen. J Exp Med (2006) 203(12):2727–35. 10.1084/jem.20061864 17116738 PMC2118158

[32] Liston A Lesage S Wilson J Peltonen L Goodnow CC. Aire regulates negative selection of organ-specific T cells. Nat Immunol (2003) 4(4):350–4. 10.1038/ni906 12612579

[33] Laan M Kisand K Kont V Moll K Tserel L Scott HS Autoimmune regulator deficiency results in decreased expression of CCR4 and CCR7 ligands and in delayed migration of CD4+ thymocytes. J Immunol (2009) 183(12):7682–91. 10.4049/jimmunol.0804133 19923453 PMC2795747

[34] Lei Y Ripen AM Ishimaru N Ohigashi I Nagasawa T Jeker LT Aire-dependent production of XCL1 mediates medullary accumulation of thymic dendritic cells and contributes to regulatory T cell development. J Exp Med (2011) 208(2):383–94. 10.1084/jem.20102327 21300913 PMC3039864

[35] Gallegos AM Bevan MJ. Central tolerance: good but imperfect. Immunol Rev (2006) 209:290–6. 10.1111/j.0105-2896.2006.00348.x 16448550

[36] Nagamine K Peterson P Scott HS Kudoh J Minoshima S Heino M Positional cloning of the APECED gene. Nat Genet (1997) 17(4):393–8. 10.1038/ng1297-393 9398839

[37] Arstila TP Jarva H. Human APECED; a sick thymus syndrome? Front Immunol (2013) 4:313. 10.3389/fimmu.2013.00313 24109480 PMC3791424

[38] Kluger N Jokinen M Krohn K Ranki A. Gastrointestinal manifestations in APECED syndrome. J Clin Gastroenterol (2013) 47(2):112–20. 10.1097/MCG.0b013e31827356e1 23314667

[39] Perheentupa J. Autoimmune polyendocrinopathy-candidiasis-ectodermal dystrophy. J Clin Endocrinol Metab (2006) 91(8):2843–50. 10.1210/jc.2005-2611 16684821

[40] Ward L Paquette J Seidman E Huot C Alvarez F Crock P Severe autoimmune polyendocrinopathy-candidiasis-ectodermal dystrophy in an adolescent girl with a novel AIRE mutation: response to immunosuppressive therapy. J Clin Endocrinol Metab (1999) 84(3):844–52. 10.1210/jcem.84.3.5580 10084559

[41] Venanzi ES Melamed R Mathis D Benoist C. The variable immunological self: genetic variation and nongenetic noise in Aire-regulated transcription. Proc Natl Acad Sci U S A (2008) 105(41):15860–5. 10.1073/pnas.0808070105 18838677 PMC2572942

[42] Malchow S Leventhal DS Lee V Nishi S Socci ND Savage PA. Aire enforces immune tolerance by directing autoreactive T cells into the regulatory T cell lineage. Immunity (2016) 44(5):1102–13. 10.1016/j.immuni.2016.02.009 27130899 PMC4871732

[43] Malchow S Leventhal DS Nishi S Fischer BI Shen L Paner GP Aire-dependent thymic development of tumor-associated regulatory T cells. Science (2013) 339(6124):1219–24. 10.1126/science.1233913 23471412 PMC3622085

[44] Yang S Fujikado N Kolodin D Benoist C Mathis D. Immune tolerance. Regulatory T cells generated early in life play a distinct role in maintaining self-tolerance. Science (2015) 348(6234):589–94. 10.1126/science.aaa7017 25791085 PMC4710357

[45] Romagnoli P Ribot J Tellier J van Meerwijk J Thymic and peripheral generation of CD4+Foxp3+ regulatory T cells. In: Jiang S, editor. Regulatory T Cells and Clinical Application. New York, NY: Springer Science + Business Media (2008). p. 29–55.

[46] Shevach EM Thornton AM. tTregs, pTregs, and iTregs: similarities and differences. Immunol Rev (2014) 259(1):88–102. 10.1111/imr.12160 24712461 PMC3982187

[47] Haribhai D Lin W Edwards B Ziegelbauer J Salzman NH Carlson MR A central role for induced regulatory T cells in tolerance induction in experimental colitis. J Immunol (2009) 182(6):3461–8. 10.4049/jimmunol.0802535 19265124 PMC2763205

[48] Haribhai D Williams JB Jia S Nickerson D Schmitt EG Edwards B A requisite role for induced regulatory T cells in tolerance based on expanding antigen receptor diversity. Immunity (2011) 35(1):109–22. 10.1016/j.immuni.2011.03.029 21723159 PMC3295638

[49] Josefowicz SZ Niec RE Kim HY Treuting P Chinen T Zheng Y Extrathymically generated regulatory T cells control mucosal TH2 inflammation. Nature (2012) 482(7385):395–9. 10.1038/nature10772 22318520 PMC3485072

[50] Fletcher AL Lukacs-Kornek V Reynoso ED Pinner SE Bellemare-Pelletier A Curry MS Lymph node fibroblastic reticular cells directly present peripheral tissue antigen under steady-state and inflammatory conditions. J Exp Med (2010) 207(4):689–97. 10.1084/jem.20092642 20308362 PMC2856033

[51] Gardner JM Devoss JJ Friedman RS Wong DJ Tan YX Zhou X Deletional tolerance mediated by extrathymic Aire-expressing cells. Science (2008) 321(5890):843–7. 10.1126/science.1159407 18687966 PMC2532844

[52] Poliani PL Kisand K Marrella V Ravanini M Notarangelo LD Villa A Human peripheral lymphoid tissues contain autoimmune regulator-expressing dendritic cells. Am J Pathol (2010) 176(3):1104–12. 10.2353/ajpath.2010.090956 20093495 PMC2832133

[53] Gardner JM Metzger TC McMahon EJ Au-Yeung BB Krawisz AK Lu W Extrathymic Aire-expressing cells are a distinct bone marrow-derived population that induce functional inactivation of CD4(+) T cells. Immunity (2013) 39(3):560–72. 10.1016/j.immuni.2013.08.005 23993652 PMC3804105

[54] Vuddamalay Y Attia M Vicente R Pomie C Enault G Leobon B Mouse and human CD8(+) CD28(low) regulatory T lymphocytes differentiate in the thymus. Immunology (2016) 148(2):187–96. 10.1111/imm.12600 26924728 PMC4863570

[55] Hale JS Fink PJ. Back to the thymus: peripheral T cells come home. Immunol Cell Biol (2009) 87(1):58–64. 10.1038/icb.2008.87 19030016 PMC2679673

[56] Thiault N Darrigues J Adoue V Gros M Binet B Perals C Peripheral regulatory T lymphocytes recirculating to the thymus suppress the development of their precursors. Nat Immunol (2015) 16(6):628–34. 10.1038/ni.3150 25939024

[57] Yu W Nagaoka H Jankovic M Misulovin Z Suh H Rolink A Continued RAG expression in late stages of B cell development and no apparent re-induction after immunization. Nature (1999) 400(6745):682–7. 10.1038/23287 10458165

[58] Borgulya P Kishi H Uematsu Y von Boehmer H. Exclusion and inclusion of alpha and beta T cell receptor alleles. Cell (1992) 69(3):529–37. 10.1016/0092-8674(92)90453-J 1316241

[59] McCaughtry TM Wilken MS Hogquist KA. Thymic emigration revisited. J Exp Med (2007) 204(11):2513–20. 10.1084/jem.20070601 17908937 PMC2118501

[60] Ben-David H Sharabi A Dayan M Sela M Mozes E. The role of CD8+CD28 regulatory cells in suppressing myasthenia gravis-associated responses by a dual altered peptide ligand. Proc Natl Acad Sci U S A (2007) 104(44):17459–64. 10.1073/pnas.0708577104 17956982 PMC2077278

[61] Liu Z Tugulea S Cortesini R Suciu-Foca N. Specific suppression of T helper alloreactivity by allo-MHC class I-restricted CD8+CD28- T cells. Int Immunol (1998) 10(6):775–83. 10.1093/intimm/10.6.775 9678758

[62] Filaci G Fravega M Negrini S Procopio F Fenoglio D Rizzi M Nonantigen specific CD8+ T suppressor lymphocytes originate from CD8+CD28- T cells and inhibit both T-cell proliferation and CTL function. Hum Immunol (2004) 65(2):142–56. 10.1016/j.humimm.2003.12.001 14969769

[63] Yu Y Zitzner JR Houlihan J Herrera N Xu L Miller J Common gamma chain cytokines promote rapid in vitro expansion of allo-specific human CD8+ suppressor T cells. PLoS One (2011) 6(12):e28948. 10.1371/journal.pone.0028948 22194954 PMC3237561

[64] Wang H Daniel V Sadeghi M Opelz G. Differences in the induction of induced human CD4(+) CD25(+) FoxP3(+) T-regulatory cells and CD3(+) CD8(+) CD28(-) T-suppressor cells subset phenotypes in vitro: comparison of phorbol 12-myristate 13-acetate/ionomycin and phytohemagglutinin stimulation. Transplant Proc (2013) 45(5):1822–31. 10.1016/j.transproceed.2012.10.061 23769052

[65] Chen L Xu Z Chang C Ho S Liu Z Vlad G Allospecific CD8 T suppressor cells induced by multiple MLC stimulation or priming in the presence of ILT3.Fc have similar gene expression profiles. Hum Immunol (2014) 75(2):190–6. 10.1016/j.humimm.2013.10.004 24220571

[66] Kim-Schulze S Scotto L Vlad G Piazza F Lin H Liu Z Recombinant Ig-like transcript 3-Fc modulates T cell responses via induction of Th anergy and differentiation of CD8+ T suppressor cells. J Immunol (2006) 176(5):2790–8. 10.4049/jimmunol.176.5.2790 16493035

[67] Scotto L Naiyer AJ Galluzzo S Rossi P Manavalan JS Kim-Schulze S Overlap between molecular markers expressed by naturally occurring CD4+CD25+ regulatory T cells and antigen specific CD4+CD25+ and CD8+CD28- T suppressor cells. Hum Immunol (2004) 65(11):1297–306. 10.1016/j.humimm.2004.09.004 15556680

[68] Liu Q Zheng H Chen X Peng Y Huang W Li X Human mesenchymal stromal cells enhance the immunomodulatory function of CD8(+)CD28(-) regulatory T cells. Cell Mol Immunol (2015) 12(6):708–18. 10.1038/cmi.2014.118 25482073 PMC4716622

[69] Chang CC Ciubotariu R Manavalan JS Yuan J Colovai AI Piazza F Tolerization of dendritic cells by T(S) cells: the crucial role of inhibitory receptors ILT3 and ILT4. Nat Immunol (2002) 3(3):237–43. 10.1038/ni760 11875462

[70] Assadiasl S Ahmadpoor P Nafar M Lessan Pezeshki M Pourrezagholi F Parvin M Regulatory T cell subtypes and TGF-beta1 gene expression in chronic allograft dysfunction. Iran J Immunol (2014) 11(3):139–52. 25266000 10.22034/iji.2014.16775

[71] Nikoueinejad H Amirzargar A Sarrafnejad A Einollahi B Nafar M Ahmadpour P Dynamic changes of regulatory T cell and dendritic cell subsets in stable kidney transplant patients: a prospective analysis. Iran J Kidney Dis (2014) 8(2):130–8. 24685736

[72] Barbon CM Davies JK Voskertchian A Kelner RH Brennan LL Nadler LM Alloanergization of human T cells results in expansion of alloantigen-specific CD8(+) CD28(-) suppressor cells. Am J Transplant (2014) 14(2):305–18. 10.1111/ajt.12575 24410845

[73] Wang Z Ouyang L Liang Z Chen J Yu Q Jeza VT CD8(low)CD28(-) T cells: a human CD8 T-suppressor subpopulation with alloantigen specificity induced by soluble HLA-A2 dimer in vitro. Cell Transplant (2015) 24(10):2129–42. 10.3727/096368914X683575 25199103

[74] Crucian B Dunne P Friedman H Ragsdale R Pross S Widen R. Alterations in levels of CD28-/CD8+ suppressor cell precursor and CD45RO+/CD4+ memory T lymphocytes in the peripheral blood of multiple sclerosis patients. Clin Diagn Lab Immunol (1995) 2(2):249–52. 7697540 10.1128/cdli.2.2.249-252.1995 PMC170139

[75] Mikulkova Z Praksova P Stourac P Bednarik J Strajtova L Pacasova R Numerical defects in CD8+CD28- T-suppressor lymphocyte population in patients with type 1 diabetes mellitus and multiple sclerosis. Cell Immunol (2010) 262(2):75–9. 10.1016/j.cellimm.2010.02.002 20219185

[76] Kouchaki E Salehi M Reza Sharif M Nikoueinejad H Akbari H. Numerical status of CD4(+)CD25(+)FoxP3(+) and CD8(+)CD28(-) regulatory T cells in multiple sclerosis. Iran J Basic Med Sci (2014) 17(4):250–5. 24904717 PMC4046232

[77] Vianna P Mondadori AG Bauer ME Dornfeld D Chies JA. HLA-G and CD8+ regulatory T cells in the inflammatory environment of pre-eclampsia. Reproduction (2016) 152(6):741–51. 10.1530/REP-15-0608 27651521

[78] Chen C Chen D Zhang Y Chen Z Zhu W Zhang B Changes of CD4+CD25+FOXP3+ and CD8+CD28- regulatory T cells in non-small cell lung cancer patients undergoing surgery. Int Immunopharmacol (2014) 18(2):255–61. 10.1016/j.intimp.2013.12.004 24345703

[79] Filaci G Fenoglio D Fravega M Ansaldo G Borgonovo G Traverso P CD8+ CD28- T regulatory lymphocytes inhibiting T cell proliferative and cytotoxic functions infiltrate human cancers. J Immunol (2007) 179(7):4323–34. 10.4049/jimmunol.179.7.4323 17878327

[80] Zhang S Ke X Zeng S Wu M Lou J Wu L Analysis of CD8+ Treg cells in patients with ovarian cancer: a possible mechanism for immune impairment. Cell Mol Immunol (2015) 12(5):580–91. 10.1038/cmi.2015.57 26166762 PMC4579658

[81] Okou DT Mondal K Faubion WA Kobrynski LJ Denson LA Mulle JG Exome sequencing identifies a novel FOXP3 mutation in a 2-generation family with inflammatory bowel disease. J Pediatr Gastroenterol Nutr (2014) 58(5):561–8. 10.1097/MPG.0000000000000302 24792626 PMC4277865

[82] Ueno A Jijon H Chan R Ford K Hirota C Kaplan GG Increased prevalence of circulating novel IL-17 secreting Foxp3 expressing CD4+ T cells and defective suppressive function of circulating Foxp3+ regulatory cells support plasticity between Th17 and regulatory T cells in inflammatory bowel disease patients. Inflamm Bowel Dis (2013) 19(12):2522–34. 10.1097/MIB.0b013e3182a85709 24097227

[83] Alroqi FJ Chatila TAT Regulatory cell biology in health and disease. Curr Allergy Asthma Rep (2016) 16(4):27 10.1007/s11882-016-0606-9 26922942 PMC5218767

[84] Buckner JH. Mechanisms of impaired regulation by CD4(+)CD25(+)FOXP3(+) regulatory T cells in human autoimmune diseases. Nat Rev Immunol (2010) 10(12):849–59. 10.1038/nri2889 21107346 PMC3046807

[85] Filaci G Fenoglio D Indiveri F. CD8(+) T regulatory/suppressor cells and their relationships with autoreactivity and autoimmunity. Autoimmunity (2011) 44(1):51–7. 10.3109/08916931003782171 20670118

[86] Joosten SA Ottenhoff TH. Human CD4 and CD8 regulatory T cells in infectious diseases and vaccination. Hum Immunol (2008) 69(11):760–70. 10.1016/j.humimm.2008.07.017 18835413

[87] Brimnes J Allez M Dotan I Shao L Nakazawa A Mayer L. Defects in CD8+ regulatory T cells in the lamina propria of patients with inflammatory bowel disease. J Immunol (2005) 174(9):5814–22. 10.4049/jimmunol.174.9.5814 15843585

